# Synthesis of New 2-(2´-Hydroxyaryl)benzotriazoles and Evaluation of Their Photochemical Behavior as Potential UV-Filters

**DOI:** 10.3390/molecules15096205

**Published:** 2010-09-03

**Authors:** Renata Farkas, Virginie Lhiaubet-Vallet, Jordi Corbera, Mercedesz Törincsi, Olga Gorchs, Carles Trullas, Oscar Jiménez, Miguel A. Miranda, Lajos Novak

**Affiliations:** 1 Research Group for Alkaloid Chemistry, Department of Organic Chemistry and Technology, Budapest University of Technology and Economics, Hungarian Academy of Sciences. Gellért tér 4, 1111 Budapest, Hungary; 2 Instituto de Tecnología Química UPV-CSIC, Universidad Politécnica de Valencia, Avda de Los Naranjos s/n, 46022 Valencia, Spain; 3 ESTEVE. Avda. Mare de Déu de Montserrat, 221, 08041 Barcelona, Spain; 4 ISDIN. Provençals 33, 08019 Barcelona, Spain

**Keywords:** benzotriazoles, benzenediol, UV-filters, UV-stabilizers

## Abstract

Two new 2-(2´-hydroxyaryl)benzotriazole derivatives were synthesized and studied by photophysical and photochemical techniques in order to assess their ability to act as UV-filters. The absorption and emission properties of both compounds were determined in solvents of different polarity. In non polar solvent, a photoinduced excited state intramolecular proton transfer was established leading to efficient non radiative dissipation of UV-energy. In addition, the compounds considered were photostable under irradiation with simulated sunlight.

## 1. Introduction

A number of molecules with intramolecular H-bonds are commercially employed as UV-stabilizers, due to their strong UV-absorption bands, short excited state lifetimes, and high photostability. An essential property of UV absorbers is that they possess a mechanism for rapid dissipation of the absorbed radiation *via* an appropriate intramolecular rearrangement. Among the best known chromophores of this type are 2-(2´-hydroxyphenyl)benzotriazole derivatives, and especially 2-(2´-hydroxy-5´-methylphenyl)benzotriazole (TIN, trade name Tinuvin P) [1], widely used as UV-stabilizer, or its silylated analog (trade name Mexoryl XL) used as a UV filter [2,3]. The photostability of these compounds has been attributed to photoinduced excited state intramolecular proton transfer (ESIPT) leading to an efficient (more than 99%) and rapid non-radiative dissipation of the harmful UV-energy.

**Scheme 1:**
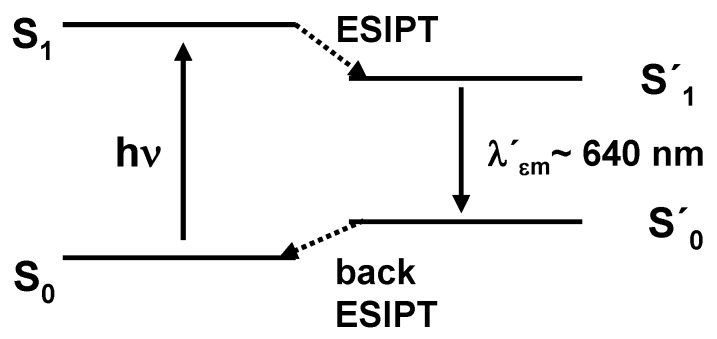
Non-radiative dissipation of UV-energy by photoinduced excited state intramolecular proton transfer.

From a mechanistic point of view, the relaxation mechanism involves ESIPT from the S_1_ state occurring in the femtosecond timescale [4,5], then radiative decay of the excited keto tautomer (fluorescence emission with a large Stokes shift) is followed by back proton transfer, causing a return to the ground state phenol (or enol) form (Scheme 1).

**Figure 1:**
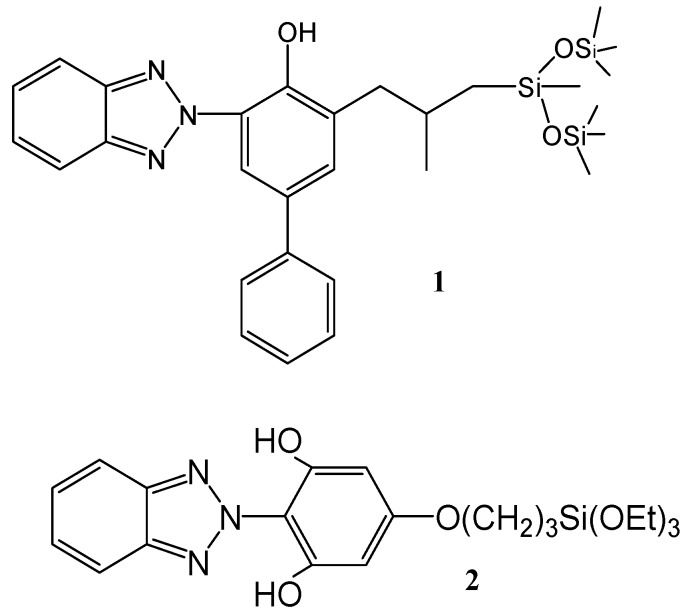
Compounds prepared and investigated.

In this context, the existence of an intramolecular hydrogen bond is of fundamental importance for photostability [6]. In the case of TIN, blue fluorescence of the non proton transferred form S_1_ (λ_max_ *ca.* 390 nm) and phosphorescence at low temperature have been observed in polar solvents [7,8,9], where the intramolecular hydrogen bond is broken in favor of the intermolecular interactions with the solvent, or when the molecule is distorted or held a non planar conformation [10,11]. In addition, a short-lived triplet state has been detected for TIN in DMSO by laser flash photolysis experiments performed at room temperature [12]. By contrast, in non polar solvents blue emission is not observed due to the strong intramolecular hydrogen bond, which favors formation of the S_1_´ excited state whose long wavelength emission is detected at low temperature [7,13]. With this background, the aim of the present study is to synthesize two derivatives of 2-(2´-hydroxyphenyl)benzotriazole ((Figure 1) and to study their photophysical and photochemical properties in order to evaluate their ability to act as new UV-filters.

## 2. Results and Discussion

### 2.1. Synthetic study

**Scheme 2:**
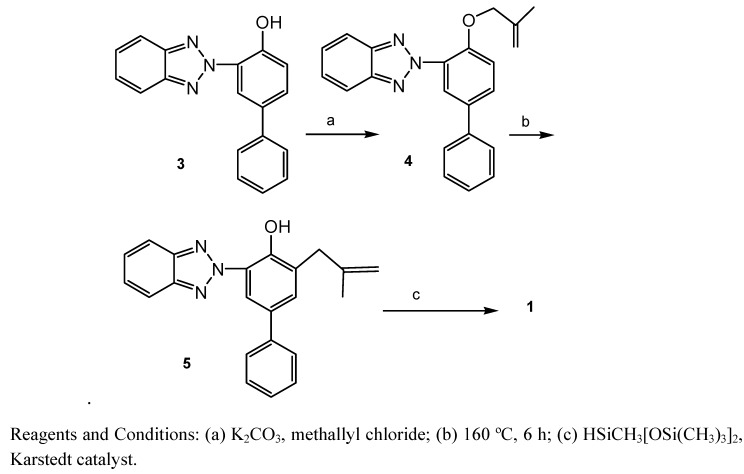
Synthesis of **1**.

Compound **1** was synthesized by a stepwise synthetic protocol shown in Scheme 2. Namely, compound **3** [14] was heated with methallyl chloride in the presence of K_2_CO_3_ and KI to afford ether **4**. Thermal rearrangement of the latter in *N*,*N*-diethylaniline afforded intermediate **5**, which was then silylated using heptamethyltrisiloxane and Karlstedt catalyst [15,16] to give the wanted product **1** in good yield.

**Scheme 3:**
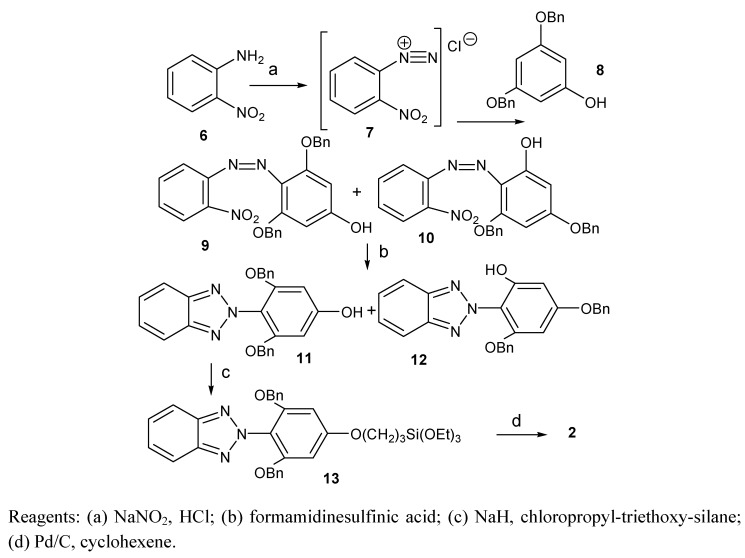
Synthesis of **2**.

Compound **2** was prepared according to Scheme 3. Commercially available *o*-nitroaniline was treated with a mixture of NaNO_2_ and HCl, and the formed diazo compound **7** was coupled with dibenzyloxyphenol (**8**) [17]. This reaction gave a 3:1 mixture of isomers **9** and **10**. Without separation the mixture was treated with formamidinesulfinic acid and the formed benzotriazoles **11** and **12** were separated by fractional crystallization and column chromatography. Compound **11** was subsequently reacted with NaH and chloropropyl-triethoxy-silane to yield compound **13**, from which the benzyl protective groups were removed by heating with Pd/C and cyclohexene. Product **2** was purified by column chromatography.

### 2.2. Absorption spectra

The absorption spectra of compound **2** in ethanol showed two maxima, λ_1_ at *ca.* 285 nm and λ_2_ at *ca.* 350 nm. As shown in Figure 1, their relative intensity was dependent on the solvent. For example, in hexane, diethyl ether or acetonitrile, only the UVA absorption band was observed, while in DMSO or DMF the band at 285 nm was predominating. These results are consistent with the data reported for the chelated and non chelated forms of TIN and other 2´-hydroxybenzotriazole derivatives [7,9]. Indeed, in solvents with low hydrogen bonding capability, the intramolecular hydrogen bond is intact and only absorption of the chelated form is observed; by contrast, in solvents where the intramolecular hydrogen bond is partially disrupted, absorption of the two forms is clearly visible.

**Figure 2:**
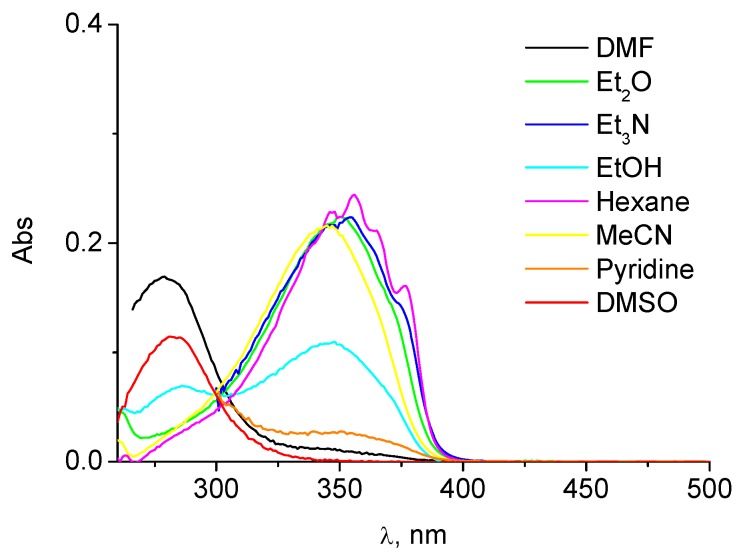
Absorption spectra of **2** in different solvents (concentration *ca.* 10^-5^ M).

**Figure 3:**
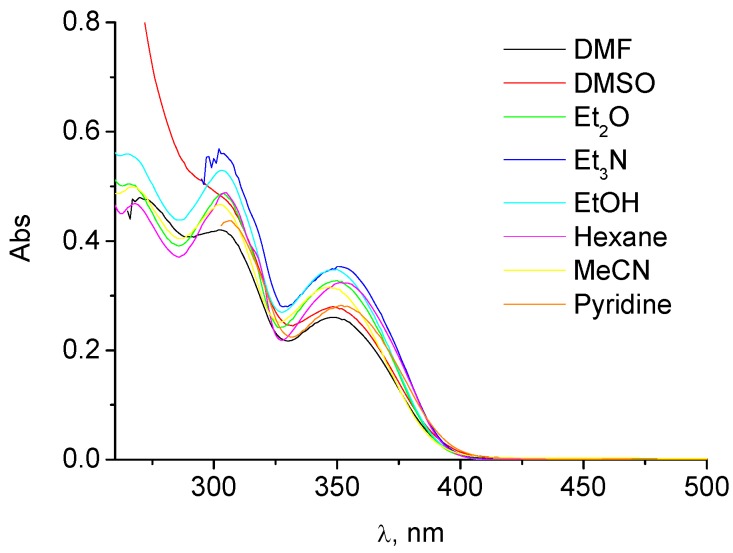
Absorption spectra of **1** in different solvents (concentration *ca.* 3 × 10^-5^ M).

In the case of compound **1**, two absorption maxima, λ_1_ at *ca.* 300 nm and λ_2_ at *ca.* 350 nm were also observed; however, by contrast with compound **2** the solvent-dependent spectral changes were not clearcut (Figure 3). This is probably attributable to overlapping of the absorption bands of the non chelated form and the hydroxybiphenyl chromophore.

### 2.3. Fluorescence emission

**Figure 4:**
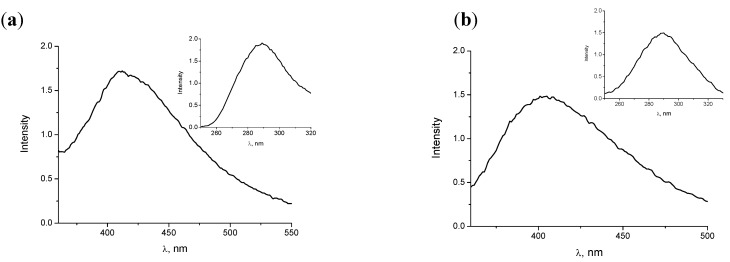
Fluorescence emission (λ_exc_ =280 nm) spectra of **2** in DMSO (a) and in ethanol(b). Inset: corresponding excitation spectra.

For compound **2**, no emission was observed at room temperature in hexane (data not shown) after excitation at 350 nm. This is in agreement with previous results reporting only a weak red fluorescence at low temperature for TIN and other 2´-hydroxybenzotriazole derivatives [7]. Conversely, in polar solvents after excitation at 280 nm, a blue fluorescence appeared at 410 nm (Figure 4a and Figure 4b). The corresponding excitation spectra nearly matched the short wavelength absorption band. Thus, taking into account the literature data and the Stokes shift (9,800 and 10,400 cm^-1^ in ethanol and DMSO, respectively), this emission was attributed to the non chelated form of compound **2**.

**Figure 5:**
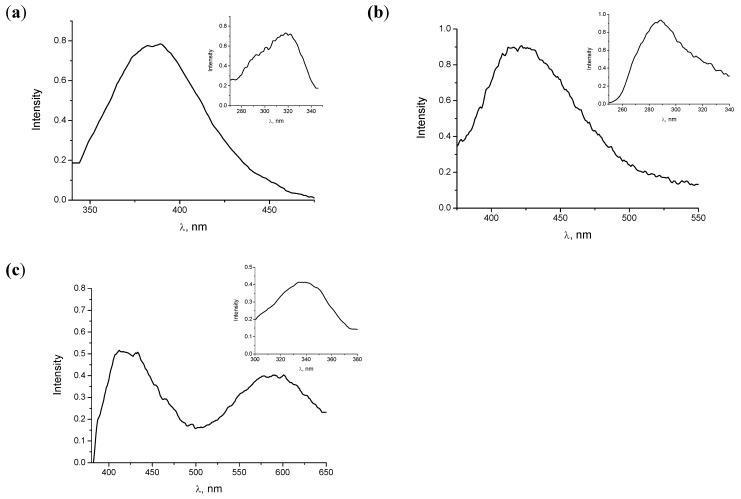
Fluorescence emission spectra of **1** in hexane at λ_exc_ =300 nm (a) andin DMSO λ_exc_ =280 nm (b) and 340 nm (c). Inset: corresponding excitation spectra.

In the case of the biphenyl derivative **1**, an emission band centred at 390 nm was detected in hexane after excitation at 300 nm; accordingly, the corresponding excitation spectrum exhibited a maximum at *ca.* 300 nm (Figure 5a). By contrast, no signal was observed after excitation at 350 nm. In ethanol, no significant fluorescence was detected after excitation at either of the two wavelengths. In the case of DMSO, a band centred at 410 nm was observed after excitation at 280 nm (Figure 5b), after excitation at 340 nm this emission was still observed and, remarkably, a new emission band at 590 nm appeared (Figure 5c). The excitation spectrum of the latter agreed well with the red shifted fluorescence of the chelated form previously described in the literature [7]. 

### 2.4. Photostability

**Figure 6:**
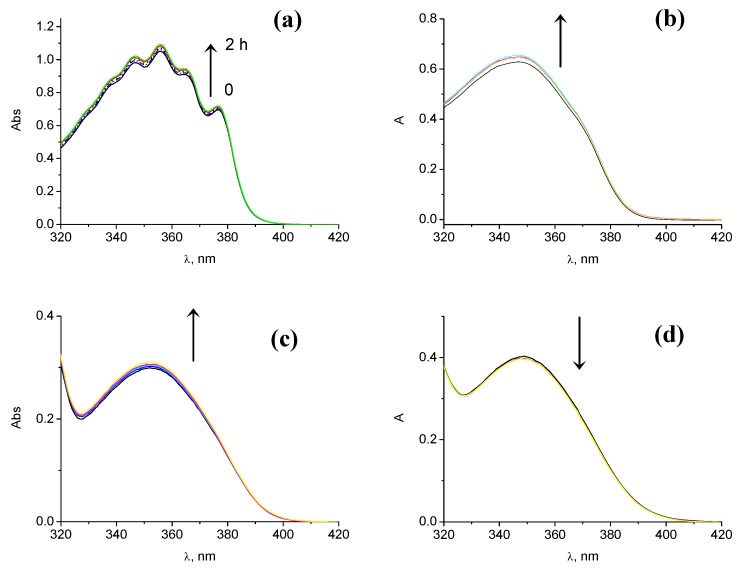
UVA-absorption changes after simulated solar irradiation (up to 2 h) of compound **2** in hexane (a) and ethanol (b). Same experiments with compound **1** in hexane **(c)** and ethanol **(d)**.

In order to assess the photostability of compounds **1** and **2**, irradiations of 2.7 × 10^-5^ M solutions were performed in different solvents and their degradation was followed by UV-Vis spectroscopy. After 2 hours of irradiation, the UVA-band was decreased by less than 5% (Figure 6). Thus, these results are similar to those previously reported for TIN and reflect the conservation of the UVA absorption capability of both compounds, an important property of UV-filters.

## 3. Experimental

### 3.1. General

Solvents for synthesis were used as received from commercial suppliers, and no further attempts were made to purify or dry them. Melting points were determined on a Büchi apparatus and are uncorrected. IR spectra were recorded on Bruker Alpha FT Spectrophotometer. ^1^H-NMR and ^13^C- NMR spectra were obtained on a Bruker DRX-500 spectrometer operating at 500 MHz and 125 MHz, respectively. All NMR spectra are reported in ppm relative to TMS. MS spectra were conducted on Agilent 6140 quadrupole LC/MS instrument. Merck precoated silica gel 60 F_254_ plates were used for TLC and Kieselgel 60 for column chromatography. 

### 3.2. Synthesis

*2-{4-[(-2-Methyl-2-propen-1-yl)oxy]-3-biphenylyl}-2H-1,2,3-benzotriazole* (**4**). Under an argon atmosphere, a stirred mixture of **3 **(5.0 g, 17 mmol), methallyl chloride (2.7 g, 30 mmol), KI (5.8 g, 35 mmol), and K_2_CO_3_ (2.4 g, 17 mmol) in butan-2-one (100 mL) was refluxed for 12 h. After cooling the precipitate was removed by filtration, the filtrate was concentrated *in vacuo*, and the resulting solid was subjected to column chromatography to give a white-yellow solid. Yield: 65% (3.77 g). m.p. 94-96 ºC. *R_f_* = 0.6 (hexane-acetone 5:2). IR (KBr): 1620, 1540, 1500, 1360, 1300, 1280, 1260 cm^-1^. ^1^H-NMR (CDCl_3_): 1.71 (s, 3H, CH_3_), 4.55 (s, 2H, C_1”_-H), 4.90 (s, 1H, C_3”_-H), 5.00 (s, 1H, C_3”_-H), 7.19 (d, *J* = 8.7 Hz, 1H, C_5’_-H), 7.33 (t, *J* = 8.5 Hz, 1H, C_10’_-H), 7.43 (m, 2H, C_9’_-H and C_11’_-H), 7.45 (m, 2H, C_5_-H and C_6_-H), 7.60 (d, *J* = 8.5 Hz, 2H, C_8’_-H and C_12’_-H), 7.70 (dd, *J* = 8.7 and 2.3 Hz, 1H, C_6’_-H), 7.94 (d, *J* = 2.3 Hz, 1H, C_2’_-H), 7.98 (m, 2H, C_4_-H and C_7_-H). ^13^C-NMR (CDCl_3_): 19.17 (CH_3_), 72.78 (C-1”), 112.90 (C-3”), 114.72 (C-5’), 118.44 (C-4 and C-7), 126.12 (C- 2’), 126.82 (C-5, C-6, C-8’, and C-12’), 127.37 (C-10’), 128.87 (C-9’ and C-11’), 129.28 (C-6’), 130.91 (C-3’), 134.31 (C-1’), 139.33 (C-7’), 139.87 (C-2”), 144.85 (C-3a and C-7a), 152.00 (C-4’). Anal. Calcd for C_22_H_19_N_3_O: C, 77.40; H, 5.61; N, 12.31. Found: C, 77.14; H, 5.72; N, 12.07.

*3-(2H-1,2,3-Benzotriazol-2-yl)-5-(2-methyl-2-propen-1-yl)-4-biphenylol* (**5**). A solution of ether **4** (10.2 g, 30 mmol) in *N,N*-diethylaniline (50 mL) was heated at reflux for 4 h. After cooling, HCl (15%, 50 mL) was added and the resulting solution was extracted with CH_2_Cl_2_ (3 × 50 mL). The combined organic extracts were washed with water, evaporated *in vacuo*, and the residue was recrystallized from hexane. Yield: 87% (8.9 g, light yellow crystals). m.p. 115-117 ºC. *Rf* = 0.8 (hexane-acetone 5:2). IR (KBr): 3400, 3100, 1620, 1500, 1480, 1450, 1360, 1280 cm^-1^. ^1^H-NMR (CDCl_3_): 1.84 (s, 3H, CH_3_), 3.58 (s, 2H, C_1”_-H), 4.80 (s, 1H, C_3”_-H), 4.89 (s, 1H, C_3”_-H), 7.35 (t, *J* = 7.5 Hz, C_10_-H), 7.46 (m, 3H, C_6_-H, C_9_-H, and C_11_-H), 7.49 (m, 2H, C_5’_-H and C_6’_-H), 7.67 (d, *J* = 8.5 Hz, 2H, C_8_-H and C_12_-H), 7.94 (m, 2H, C_4’_-H and C_7’_-H), 8.56 (d, *J* = 2.3 Hz, 1H, C_2_-H), 11.67 (s, 1H, OH). ^13^C-NMR (CDCl_3_): 22.57 (CH_3_), 38.07 (C-2”), 111.92 (C-3”), 117.67 (C-4’ and C-7’), 117.75 (C-2), 125.39 (C-3), 126.83 (C-8 and C-12), 127.14 (C-10), 127.74 (C-5’ and C-6’), 128.81 (C-9 and C-11), 129.91 (C-6), 130.10 (C-5), 132.67 (C-1), 139.96 (C-7), 142.82 (C-3a’ and C-7a’), 144.25 (C-2”), 147.52 (C-4). Anal. Calcd for C_22_H_19_N_3_O: C, 77.40; H, 5.61; N, 12.31. Found: C, 77.17; H, 5.82; N, 12.17.

*3-(2H-1,2,3-Benzotriazol-2-yl)-5-(2-methyl-3-{1,3,3,3-tetramethyl-1-[(trimethylsilyl)oxy]disiloxanyl}- propyl)-4-biphenylol* (**1**). To a stirred solution of **5** (2.0 g, 6 mmol) in dry xylene (40 mL) was added 1,1,1,3,5,5,5-heptamethyltrisiloxane (2.5 mL, 2.2 g, 9 mmol) and Karlstedt catalyst (15 drops) and the resulting mixture was heated at 100 ºC for 5 h. After cooling, the solvent was evaporated *in vacuo* and the residue was purified by column chromatography to give compound **1**. Yield: 72% (2.4 g), yellow oil. *R_f_* = 0.63 (hexane-acetone 5:02). IR (KBr): 3250 (OH), 1625, 1500, 1250, 1090, 1060 cm^-1^. ^1^H- NMR (CDCl_3_): 0.10 (s, 3H, CH_3_), 0.11 (s, 9H, 3CH_3_), 0.12 (s, 9H, CH_3_), 0.56 (dd, *J* = 14.8 and 9.2 Hz, 1H, C_3”_-H), 0.78 (dd, *J* = 14.8 and 4.7 Hz, 1H, C_3”_-H), 1.05 (d, *J* = 6.6 Hz, 3H, CH_3_), 2.23 (m, 1H, C_2”_-H), 2.73 (dd, *J* = 13.2 and 8.2 Hz, 1H, C_1”_-H), 2.89 (dd, *J* = 13.2 and 6.2 Hz, 1H, C_1”_-H), 7.38 (t, *J* = 7.4 Hz, 1H, C_10_-H), 7.49 (m, 3H, C_6_-H, C_9_-H, and C_11_-H), 7.52 (m, 2H, C_5’_-H and C_6’_-H), 7.71 (d, *J* = 7.5 Hz, 2H, C_8_-H and C_12_-H), 7.98 (m, 2H, C_4’_-H and C_7’_-H), 8.57 (d, *J* = 3.3 Hz, 1H, C_2_-H), 11.60 (s, 1H, OH). ^13^C-NMR (CDCl_3_): 0.85 (CH_3_), 1.87 (6CH_3_), 22.48 (CH_3_), 26.18 (C-3”), 29.31 (C-2”), 41.27 (C-1”), 117.39 (C-2), 117.66 (C-4’ and C-7’), 125.34 (C-3), 126.83 (C-8 and C-12), 127.03 (C-10), 127.64 (C-5’ and C-6’), 128.77 (C-9 and C-11), 130.56 (C-6), 132.14 (C-5), 132.34 (C-1), 140.14 (C-7), 142.81 (C-3a’ and C-7a’), 147.66 (C-4). MS (*m/z*): 563 (M^+^, 5%), 474 [M^+^- (CH_3_)_3_SiO, 46], 101 (100). Anal. Calcd for C_29_H_41_N_3_O_3_Si_3_: C, 61.77; H, 7.33; N, 7.45. Found: C, 61.70; H, 7.52; N, 7.67.

*4-(2H-1,2,3-Benzotriazol-2-yl)-3,5-bis(benzyloxy)phenol* (**11**). To a cold (0–5 ºC) suspension of 2-nitrobenzeneamine (**6**, 27.62 g, 0.2 mol) in concentrated HCl 100 mL), crashed ice (100g) and water (100 mL), was added dropwise 5N solution of NaNO_2_ (41 mL). The resulting mixture was stirred at 5 ºC for 1 h and then filtered. This solution of **7** was then added dropwise to a stirred solution of 3,5-bis(benzyloxy)phenol (**8**, 61.3 g, 0.2 mol) in methanol (600 mL). Stirring was continued at room temperature for 2 h and the precipitated red crystalline compound was collected by filtration to give a 2:1 mixture of isomeric azo compounds **9** and **10**. Yield: 88% (80.77 g). m.p. 171-180 ºC. *R_f_* = 0.52 and 0.88 (toluene-MeOH 9:1). To a boiling suspension of the above mixture of azo-compounds (45.5 g, 0.1 mol) in a mixture of EtOH (250 mL) and 4N NaOH (250 mL) was added formamidinesulfinic acid (23.65 g, 0.22 mol) during 0.5 h. The resultant mixture was heated with stirring for 1 h, cooled to room temperature, and then poured into a mixture of ice and water (700 g). The resulting mixture was acidified with concentrated HCl (80 mL) and extracted with EtOAc. The organic layer was successively washed with water and brine, and then dried (MgSO_4_). Evaporation of the solvent *in vacuo* afforded a red oil (42 g), which was then treated with a mixture of hexane and EtOAc (60 mL, each). The precipitated crystalline compounds were collected by filtration and washed with hexane-acetone (1:1) to give compound **11**. Yield: 28% (12.64 g). m.p. 147-152 ºC. *R_f_* =0.38 (toluene-MeOH 9:1) and 0.26 (hexane-EtOAc 2:1). ^1^H-NMR (CDCl_3_): 4.73 (s, 4H, 2OCH_2_), 6.11 (s, 2H, C_2_-H and C_6_-H), 6.98 (m, 4H, 2C_2”_-H and 2C_6”_-H), 7.07 (m, 2H, 2C_4”_-H), 7.10 (m, 4H, 2C_3”_-H and 2C_5”_-H), 7.43 (dd, *J* = 6.7 and 3 Hz, 2H, C_5’_-H and C_6’_-H), 7.95 (dd, *J* = 6.7 and 3 Hz, 1H, C_4’_-H and C_7’_-H), 8.62 (s, 1H, OH). ^13^C- NMR (CDCl_3_): 70.31 (OCH_2_), 94.55 (C-2), 113.03 (C-4), 118.23 (C-4’ and C-7’), 126.61 (C-2” and C-6”), 126.88 (C-5’ and C-6’), 127.65 (C-4”), 128.27 (C-3” and C-5”), 136.01 (C-1”), 144.37 (C-3a’ and C-7a’), 156.00 (C-3 and C-5), 159.99 (C-1). Anal. Calcd for C_26_H_21_N_3_O_3_: C, 73.74; H, 5.00; N, 9.92. Found: C, 73.47; H, 5.22; N, 9.67.

The isomer – *2-(2H-1,2,3-benzotriazol-2-yl)-3,5-bis(benzyloxy)phenol* (**12**) *–* was ISOLATED by column chromatography From the mother liquor. Yield: 12% (5.4 g). m.p. 71-78 ºC. *R_f _*= 0.73 (toluene-MeOH 9:1) and 0.48 (hexane-EtOAc 2:1). ^1^H-NMR (CDCl_3_): 5.04 (s, 2H, OCH_2_), 5.16 (s, 2H, OCH_2_), 6.38 (d, *J*= 2.4 Hz, 1H, C_4_-H), 6.41 (d, *J* = 2.4 Hz, 1H, C_6_-H), 7.25 (t, *J* = 7 Hz, 1H, C_4”_-H), 7.30 (t, *J* = 7.5 Hz, 2H, C_3”_-H and C_5”_-H), 7.35 (t, *J* = 7.2 Hz, 1H, C_4”’_-H), 7.39 (m, 4H, C_2”_-H ,C_6”_-H, C_3”’_-H and C_5”’_-H), 7.41 (m, 2H, C_2”’_-H and C_6”’_-H), 7.45 (dd, *J* = 6.5 Hz, 2H, C_5’_-H and C_6’_-H), 7.94 (dd, *J* = 6.5 Hz, 2H, C_4’_-H and C_7’_-H), 9.36 (br. s, 1H, OH). ^13^C-NMR (CDCl_3_): 70.33 (OCH_2_), 71.28 (OCH_2_), 95.21 (C-4), 95.81 (C-6), 112.50 (C-2), 117.87 (C-4’ and C-7’), 126.86 (C-2” and C-6”), 127.23 (C-5’ and C-6’), 127.62 (C-2”’ and C-6”’), 127.77 (C-4”), 128.24 (C-4”’), 128.41 (C-3” and C-5”), 128.68 (C-3”’ and C-5”’), 136.18 (C-1”’), 136.30 (C-1”), 143.64 (C-3a’ and C-7a’), 152.80 (C-1), 153.67 (C-3), 160.62 (C-5). Anal. Calcd for C_26_H_21_N_3_O_3_: C, 73.74; H, 5.00; N, 9.92. Found: C, 73.49; H, 5.27; N, 9.72.

*2-{2,6-bis(Benzyloxy)-4-[3-(triethoxysilyl)propoxy]phenyl}-2H-1,2,3-benzotriazole* (**13**). To a stirred suspension of NaH (1.8 g, 60% in mineral oil, 44 mmol) in dry DMF (80 mL) was added dropwise a solution of compound **11** (16.9 g, 40 mmol) in dry DMF (140 mL). After stirring for 45 min at room temperature chloropropyl-triethoxy-silane (9.87 g, 40 mmol) and KI (6.43 g, 40 mmol) were added, and the resultant mixture was heated at 80 ºC for 8 h. After cooling, the reaction mixture was poured into water (200 mL), extracted with ether (3 × 150 mL), the organic layer was washed with brine, and then dried (MgSO_4_). Evaporation of the solvent *in vacuo* afforded a reddish oil, which was purified by column chromatography (hexane-EtOAc 5:1 as eluent). Yield: 37% (9.13 g), semi solid. *R_f_* = 0.7 (hexane-EtOAc 2:1). ^1^H-NMR (CDCl_3_): 0.79 (m, 2H, CH_2_-Si), 1.27 (t, *J* =7 Hz, 9H, 3CH_3_), 1.93 (m, 2H, CH_2_), 3.88 (q, *J* = 7 Hz, 6H, 3 OCH_2_), 3.92 (t, *J* = 6.6 Hz, 2H, OCH_2_), 5.07 (s, 4H, 2OCH_2_), 6.28 (s, 2H, C_3’_-H and C_5’_-H), 7.17 (d, *J* = 7.5 Hz, 4H, C_2”_-H and C_6”_-H), 7.23 (m, 6H, C_3”_-H, C_4”_-H, and C_5”_-H), 7.44 (dd, *J* = 6.6 and 3 Hz, 2H, C_5_-H and C_6_-H), 8.1 (dd, *J* = 6.6 and 3 Hz, 2H, C_4_-H and C_7_-H). ^13^C-NMR (CDCl_3_): 6.47 (C-Si), 18.30 (CH_3_), 22.62 (C-C-Si), 58.43 (C-O),70.16 (C-O), 70.57 (C-O), 93.38 (C-3’ and C-5’), 114.53 (C-1’), 118.47 (C-4 and C-7), 126.29 (C-5 and C-6), 126.52 (C-2” and C-6”), 127.62 (C-4”), 128.32 (C-3” and C-5”), 136.19 (C-1”), 144.74 (C-3a and C-7a), 156.06 (C-2’ and C-6’), 161.65 (C-4’). Anal. Calcd for C_35_H_41_N_3_O_6_Si: C, 66.96; H, 6.58; N, 6.69. Found: C, 67.09; H, 6.29; N, 6.47.

*2-(2H-1,2,3-Benzotriazol-2-yl)-5-[3-(triethoxysilyl)propoxy]-1,3-benzenediol* (**2**). A stirred mixture of compound **13** (2 g, 3.2 mmol), palladium catalyst (1 g, 10% Pd on charcoal), and cyclohexene (64 mL, 52 g, 0.6 mol) in EtOH (80 mL) was heated at reflux for 1.5 h. After filtration, the solvent was evaporated *in vacuo* and the residue was purified by column chromatography. Yield: 76% (1.08 g, light yellow crystals). m.p. 69-74 ºC. *R_f_* = 0.27 (CH_2_Cl_2_). ^1^H-NMR (CDCl_3_): 0.80 (m, 2H, CH_2_-Si), 1.27 (t, *J* = 7 Hz, 9H, 3 CH_3_), 1.95 (m, 2H, CH_2_), 3.88 (q, *J* = 7 Hz, 6H, 3 O-CH_2_), 4.00 (t, *J* = 6.6 Hz, 2H, CH_2_), 6.29 (s, 2H, C_5_-H and C_6_-H), 7.93 (m, 2H, C_4_-H and C_7_-H), 11.56 (s, 2H, OH). ^13^C-NMR (CDCl_3_): 6.45 (C-Si), 18.29 (CH_3_), 22.64 (C-2’’), 58.43 (C-O), 70.10 (C-1’’), 95.73 (C-6), 108.17 (C-2), 116.62 (C-4’ and C-7’), 127.87 (C-5’ and C-6’), 140.18 (C-3a and C-7a), 152.07 (C-1 and C-3), 160.93 (C-5). MS (*m/z*): 447 (M^+^, 8 %), 283 [M^+^-HSi(OEt)_3_, 12], 256 (17), 124 (100). Anal. Calcd for C_21_H_29_N_3_O_6_Si: C, 56.36; H, 6.53; N, 9.39. Found: C, 56.59; H, 6.28; N, 9.49.

### 3.3. Spectroscopic studies

#### General

The solvents used were of spectroscopic grade and provided by Sigma and Merck. Absorption spectra were recorded on a double beam Varian UV-VIS Cary 300 Scan spectrometer, using 10 mm pathway quartz cuvettes. The steady-state fluorescence experiments were carried out on a Photon Technology International (PTI) LPS-220B spectrofluorometer. Emission and excitation spectra were recorded using 10 × 10 mm^2^ quartz cells with 4 mL capacity, using air-equilibrated solutions. The absorbance was adjusted at the excitation wavelength and kept in the range 0.10–0.15. All the experiments were carried out at room temperature.

### 3.4. Steady state photolysis

Photolysis of 2.7 × 10^-4^ M of compounds **1** and **2** were performed to evaluate their photostability in DMSO, EtOH and hexane. Broadband irradiations of the solutions were carried out using an ABET Technology solar simulator with a 200 W Xe arc. Its output was adequately filtered to produce a spectrum approximating natural sunlight (1.5 G air mass filter). The spectral output was measured as ca 1000 W/m^2^. The photostability was evaluated by UV-Vis spectroscopy, recording the changes of the UVA-band.

## 4. Conclusions

In this work, two new 2-(2´-hydroxyaryl)benzotriazoles were considered as potential UV-filters. The typical spectroscopic properties of 2´-hydroxybenzotriazole derivatives were obtained for compound **1**, which undergoes a photoinduced excited state intramolecular proton transfer in hexane; conversely the use of more polar solvents like ethanol or DMSO results in the disruption of the intramolecular bond. By contrast, no solvent effect was observed for compound **2**. Moreover, steady state photolysis showed that both compounds exhibit a potential applicability as UV filters due to their UVA-absorption capability and to their photostability.
